# Calibration of Magnetometers with GNSS Receivers and Magnetometer-Aided GNSS Ambiguity Fixing

**DOI:** 10.3390/s17061324

**Published:** 2017-06-08

**Authors:** Patrick Henkel

**Affiliations:** Department of Electrical and Computer Engineering, Technische Universität München, Arcisstrasse 21, 80333 München, Germany; patrick.henkel@tum.de; Tel.: +49-89-289-23462

**Keywords:** magnetometer, calibration, satellite navigation, attitude determination, carrier phase

## Abstract

Magnetometers provide compass information, and are widely used for navigation, orientation and alignment of objects. As magnetometers are affected by sensor biases and eventually by systematic distortions of the Earth magnetic field, a calibration is needed. In this paper, a method for calibration of magnetometers with three Global Navigation Satellite System (GNSS) receivers is presented. We perform a least-squares estimation of the magnetic flux and sensor biases using GNSS-based attitude information. The attitude is obtained from the relative positions between the GNSS receivers in the North-East-Down coordinate frame and prior knowledge of these relative positions in the platform’s coordinate frame. The relative positions and integer ambiguities of the periodic carrier phase measurements are determined with an integer least-squares estimation using an integer decorrelation and sequential tree search. Prior knowledge on the relative positions is used to increase the success rate of ambiguity fixing. We have validated the proposed method with low-cost magnetometers and GNSS receivers on a vehicle in a test drive. The calibration enabled a consistent heading determination with an accuracy of five degrees. This precise magnetometer-based attitude information allows an instantaneous GNSS integer ambiguity fixing.

## 1. Introduction

A precise attitude information is essential for numerous applications, e.g., for navigation, for flight control and for constructions. Magnetometers provide attitude information globally but are affected by sensor biases and measurement errors. The latter ones can be systematic due to metallic environment and/or stochastic due to measurement noise. Sensor biases and systematic errors can be removed by calibration.

The calibration can be performed with three Global Navigation Satellite System (GNSS) receivers (e.g., GPS, Galileo, GLONASS, Beidou) being placed on the same platform as the magnetometer. Satellite navigation enables the determination of the relative position between both GNSS receivers in a local coordinate frame, e.g., the North-East-Down (NED) frame. The obtained relative position is related to the prior knowledge of the relative position in the *body-fixed* frame to obtain the platform’s *attitude*.

The use of GNSS carrier phase measurements is required to obtain a *precise* attitude information. The carrier phases are periodic, which requires an integer ambiguity resolution for each differential measurement. An efficient integer least-squares estimation of these integer ambiguities has been developed by Teunissen in [1]. His ”*Least Squares Ambiguity Decorrelation Adjustment*” (LAMBDA) method includes an integer decorrelation and sequential tree search. The success rate of integer ambiguity fixing can be substantially increased by including prior information on the baseline. Teunissen has included prior information on the baseline length in [2,3] and Henkel and Günther have additionally included prior information on the attitude in [4]. The prior information on the length and attitude angles can be either a ”hard” or ”soft” information.

Henkel et al. have developed a method for determining magnetometer biases using two GNSS receivers in [5]. Crassidis et al. [6] and Psiaki et al. [7] used extended and unscented Kalman filters to estimate the sensor biases and scaling factors.

The purpose of this paper is threefold: first, we describe a method for calibration of magnetometer biases using attitude information from three GNSS receivers. Second, we describe a method for fast and reliable integer ambiguity fixing using magnetometer-based attitude information. Finally, the proposed methods are verified with measurements of low-cost magnetometers and GNSS receivers in a test drive with a vehicle.

## 2. Measurement Models

In this section, we introduce a precise model for the magnetometer measurements.

### Measurement Model for Magnetic Field Sensor

We model the magnetic flux measurement in the sensor-fixed *s*-frame at epoch $t_{n}$ as a sum of true magnetic flux, measurement bias $b_{m^{s}}$ and measurement noise $\varepsilon_{m^{s}}$:

(1) $$m^{s}\left(t_{n}\right)=R_{g}^{s}\left(t_{n}\right)\cdot m^{g}\left(t_{n}\right)+b_{m^{s}}\left(t_{n}\right)+\varepsilon_{m^{s}}\left(t_{n}\right),$$

where the magnetic flux is expressed in the local geomagnetic (g-) frame (right-hand coordinate system with the first axis pointing towards geomagnetic north pole, the second axis parallel to plane of magnetic flux, and the third axis perpendicular to magnetic flux and pointing downwards) and transformed into the sensor-fixed frame. The transformation $R_{g}^{s}$ is performed in three steps:

- transformation from local *geomagnetic* frame into local *geographic* navigation-frame, depending on the magnetic declination δν,
- transformation from local geographic navigation-frame into body-fixed frame, depending on the attitude of sensor’s platform (roll φ, pitch θ and heading ψ),
- transformation from body-fixed frame into sensor-fixed frame, depending on misalignment errors of sensors (roll offset Δφ, pitch offset Δθ and heading offset Δψ).

The transformation $R_{g}^{s}$ follows as:

(2) $$R_{g}^{s}=R_{b}^{s}\left(\Delta \varphi \left(t_{n}\right),\Delta \theta \left(t_{n}\right),\Delta \psi \left(t_{n}\right)\right)\cdot R_{n}^{b}\left(\varphi \left(t_{n}\right),\theta \left(t_{n}\right),\psi \left(t_{n}\right)\right)\cdot R_{g}^{n}\left(\Delta \nu \right),$$

with

(3) $$R_{b}^{s}=R_{1}\left(\Delta \varphi \right)R_{2}\left(\Delta \theta \right)R_{3}\left(\Delta \psi \right),$$

and

(4) $$\begin{array}{cc} R_{n}^{b}= & R_{1}\left(\varphi \right)R_{2}\left(\theta \right)R_{3}\left(\psi \right)\\ = & \left(\begin{array}{ccc} \cos(\theta )\cos(\psi ) & \cos(\theta )\sin(\psi ) & -\sin(\theta )\\ \sin(\varphi )\sin(\theta )\cos(\psi )-\cos(\varphi )\sin(\psi ) & \sin(\varphi )\sin(\theta )\sin(\psi )+\cos(\varphi )\cos(\psi ) & \sin(\varphi )\cos(\theta )\\ \cos(\varphi )\sin(\theta )\cos(\psi )+\sin(\varphi )\sin(\psi ) & \cos(\varphi )\sin(\theta )\sin(\psi )-\sin(\varphi )\cos(\psi ) & \cos(\varphi )\cos(\theta ) \end{array}\right) \end{array}$$

and

(5) $$R_{g}^{n}=R_{3}\left(\Delta \nu \right).$$

## 3. Calibration of Magnetic Flux Sensors

The calibration of the magnetic flux sensor requires the estimation of the magnitude $m^{g}$ of the magnetic flux and of the sensor bias $b_{m^{s}}$. We consider the rotation matrix $R_{g}^{s}$ as known. It is derived from GNSS in this section.

### 3.1. Estimation of Magnetometer Biases

The calibration of the magnetic flux requires measurements with *rotational dynamics* of multiple epochs to enable a *separation* of $m^{g}$ and $b_{m^{s}}$. The magnetic flux $m^{g}$ and bias $b_{m^{s}}$ can be assumed constant over a few epochs. Therefore, we stack the magnetic flux measurement of Equation (1) of *n* epochs in a column vector and express them in terms of the unknowns:

(6) $$\left(\begin{array}{c} m^{s}\left(t_{1}\right)\\ \vdots \\ m^{s}\left(t_{n}\right) \end{array}\right)=A\left(\begin{array}{c} m^{g}\\ b_{m^{s}} \end{array}\right)+\left(\begin{array}{c} \eta_{m^{s}}\left(t_{1}\right)\\ \vdots \\ \eta_{m^{s}}\left(t_{n}\right) \end{array}\right)\;\mathrm{with}\;A=\left(\begin{array}{cc} R_{g}^{s}\left(t_{1}\right) & 1\\ \vdots & \vdots \\ R_{g}^{s}\left(t_{n}\right) & 1 \end{array}\right).$$

The magnetic flux and bias are determined such that the sum of squared measurement residuals is minimized, i.e.,

(7) $$\begin{array}{ccc} \left(\begin{array}{c} {\hat{m}}^{g}\\ {\hat{b}}_{m^{s}} \end{array}\right) & = & \arg\min_{m^{g},b_{m^{s}}}{∥\left(\begin{array}{c} m^{s}\left(t_{1}\right)\\ \vdots \\ m^{s}\left(t_{n}\right) \end{array}\right)-A\left(\begin{array}{c} m^{g}\\ b_{m^{s}} \end{array}\right)∥}_{\Sigma^{-1}}^{2}\\ & = & {\left(A^{T}\Sigma^{-1}A\right)}^{-1}A^{T}\Sigma^{-1}\left(\begin{array}{c} m^{s}\left(t_{1}\right)\\ \vdots \\ m^{s}\left(t_{n}\right) \end{array}\right). \end{array}$$

### 3.2. Attitude Determination with Three GNSS Receivers

In this section, we derive the attitude information needed for calibration of the magnetometer from three GNSS receivers.

It is assumed that all of the three GNSS receivers and the magnetometer are mounted on the same platform. The first GNSS receiver serves as reference receiver for attitude determination. The reference receiver and the other two receivers span two independent *baselines* pointing from the reference receiver to the other receivers. GNSS enables the estimation of these baselines in a local North-East-Down coordinate frame. The relation of the baseline in the North-East-Down and local body-fixed coordinate frames provides the attitude information.

GNSS receivers provide two types of range measurements with the following characteristics:

- Carrier phase measurement $\lambda \varphi_{r}^{k}$,
  ⊕ carrier phase can be tracked with millimeter accuracy,
  ⊖ carrier phase is period with λ=19 cm and requires ambiguity resolution,
- Pseudorange measurement $\rho_{r}^{k}$,
  ⊕ pseudorange is an unambiguous range measurement,
  ⊖ pseudorange measurement is more sensitive to multipath,
  ⊖ pseudorange measurement can only be tracked with meter-level accuracy.

#### 3.2.1. Modeling of Differential GNSS Measurements

We use both types of measurements and perform differential measurements between the reference receiver (being indexed by 1) and any other receiver (being indexed by *r*) to eliminate atmospheric delays, orbital errors, satellite clock offsets and biases. Additionally, a reference satellite *l* is selected and the differential measurements of the reference satellite are subtracted from the differential measurements of any other satellite k∈{1,…,K} to eliminate receiver clock errors and biases. The obtained double difference carrier phase measurement is modeled as

(8) $$\begin{array}{ccc} \lambda \varphi_{1r}^{kl} & := & \left(\lambda \varphi_{1}^{k}-\lambda \varphi_{r}^{k}\right)-\left(\lambda \varphi_{1}^{l}-\lambda \varphi_{r}^{l}\right)\\ & = & {\left({\vec{e}}^{\;kl}\right)}^{T}R_{n}^{e}\left(\lambda_{1},\varphi_{1}\right){\vec{b}}_{1r}^{n}+\lambda N_{1r}^{kl}+\varepsilon_{1r}^{kl}\;\forall \;r,k,l, \end{array}$$

with the wavelength λ, the carrier phase measurement $\varphi_{r}^{k}$ in unit of cycles, the normalized line of sight vector ${\vec{e}}^{\;k}$ from satellite *k* to the receiver platform, the rotation matrix $R_{n}^{e}$ from the navigation frame into the Earth Centered Earth Fixed (ECEF) (e-) coordinate frame depending on the absolute position of the platform (given by longitude $\lambda_{1}$ and latitude $\varphi_{1}$), the integer ambiguity $N_{r}^{k}$, the phase noise $\varepsilon_{r}^{k}$ and the double difference operator ${\left(\cdot \right)}_{1r}^{kl}=\left({\left(\cdot \right)}_{1}^{k}-{\left(\cdot \right)}_{r}^{k}\right)-\left({\left(\cdot \right)}_{1}^{l}-{\left(\cdot_{r}\right)}^{l}\right)$.

Similarly, the double difference pseudorange measurement is modeled as

(9) $$\begin{array}{ccc} \rho_{1r}^{kl} & := & \left(\rho_{1}^{k}-\rho_{r}^{k}\right)-\left(\rho_{1}^{l}-\rho_{r}^{l}\right)\\ & = & {\left({\vec{e}}^{\;kl}\right)}^{T}R_{n}^{e}\left(\lambda_{1},\varphi_{1}\right){\vec{b}}_{1r}^{n}+\Delta \rho_{\mathrm{MP},1r}^{kl}+\eta_{1r}^{kl}\;\forall \;r,k,l, \end{array}$$

with the pseudorange multipath error $\Delta \rho_{\mathrm{MP},r}^{k}$ and pseudorange noise $\eta_{r}^{k}$.

#### 3.2.2. Joint Estimation of Baselines, Pseudorange Multipaths and Ambiguities

The double difference carrier phase and pseudorange measurements from both baselines are stacked in a common measurement vector:

(10) $$\begin{array}{ccc} z_{n}:=\left(\begin{array}{c} \lambda \varphi_{12}\left(t_{n}\right)\\ \lambda \varphi_{13}\left(t_{n}\right)\\ \rho_{12}\left(t_{n}\right)\\ \rho_{13}\left(t_{n}\right) \end{array}\right) & = & \underset{H_{n}}{\underset{︸}{\left(\begin{array}{cccccc} H & 0 & \lambda \cdot 1 & 0 & 0 & 0\\ 0 & H & 0 & \lambda \cdot 1 & 0 & 0\\ H & 0 & 0 & 0 & 1 & 0\\ 0 & H & 0 & 0 & 0 & 1 \end{array}\right)}}\left(\begin{array}{c} {\vec{b}}_{12}^{n}\left(t_{n}\right)\\ {\vec{b}}_{13}^{n}\left(t_{n}\right)\\ N_{12}\\ N_{13}\\ \Delta \rho_{{\mathrm{MP}}_{12}}\left(t_{n}\right)\\ \Delta \rho_{{\mathrm{MP}}_{13}}\left(t_{n}\right) \end{array}\right)\\ & & +\left(\begin{array}{cccccc} 1 & -1 & 0 & 0 & 0 & 0\\ 1 & 0 & -1 & 0 & 0 & 0\\ 0 & 0 & 0 & 1 & -1 & 0\\ 0 & 0 & 0 & 1 & 0 & -1 \end{array}\right)\left(\begin{array}{c} \varepsilon_{\lambda \varphi_{1}}\left(t_{n}\right)\\ \varepsilon_{\lambda \varphi_{2}}\left(t_{n}\right)\\ \varepsilon_{\lambda \varphi_{3}}\left(t_{n}\right)\\ \eta_{\rho_{1}}\left(t_{n}\right)\\ \eta_{\rho_{2}}\left(t_{n}\right)\\ \eta_{\rho_{3}}\left(t_{n}\right) \end{array}\right), \end{array}$$

with

(11) $$\lambda \varphi_{1r}=\left(\begin{array}{c} \lambda \varphi_{1r}^{1l}\\ \vdots \\ \lambda \varphi_{1r}^{Kl} \end{array}\right),\;\rho_{1r}=\left(\begin{array}{c} \rho_{1r}^{1l}\\ \vdots \\ \rho_{1r}^{Kl} \end{array}\right)\;\mathrm{and}\;H=\left(\begin{array}{c} {\left({\vec{e}}^{\;1l}\right)}^{T}\\ \vdots \\ {\left({\vec{e}}^{\;Kl}\right)}^{T} \end{array}\right)\cdot R_{n}^{e}\left(\lambda_{1},\varphi_{1}\right).$$

The number of unknowns of Equation (10) exceeds the number of measurements of one epoch, but the ambiguities are constant over time. Thus, measurements from multiple epochs are required to estimate the baselines, ambiguities and pseudorange multipaths. We introduce a state space/movement model to describe the temporal behavior of the state parameters, i.e.,

(12) $$\begin{array}{ccc} x_{n} & = & {\left({\left({\vec{b}}_{12}^{n}\left(t_{n}\right)\right)}^{T},{\left({\vec{b}}_{13}^{n}\left(t_{n}\right)\right)}^{T},N_{12}^{T},N_{13}^{T},{\left(\Delta \rho_{{\mathrm{MP}}_{12}}\left(t_{n}\right)\right)}^{T},{\left(\Delta \rho_{{\mathrm{MP}}_{13}}\left(t_{n}\right)\right)}^{T}\right)}^{T}\\ & = & \Phi_{n}x_{n-1}+\eta_{x_{n}}, \end{array}$$

with state transition matrix $\Phi_{n}$ and process noise $\eta_{x_{n}}$. A Kalman filter (see Brown and Hwang [8]) is used to estimate the state vector. We first disregard the integer property of ambiguities to obtain a float solution. The Kalman filter includes the state prediction

(13) $$\begin{array}{ccc} {\hat{x}}_{n}^{-} & = & \Phi_{n}{\hat{x}}_{n-1}^{+}\\ \Sigma_{{\hat{x}}_{n}^{-}} & = & \Phi_{n}\Sigma_{{\hat{x}}_{n-1}^{+}}{\left(\Phi_{n}\right)}^{T}+\Sigma_{x_{n}}, \end{array}$$

and the subsequent state update

(14) $$\begin{array}{ccc} {\hat{x}}_{n}^{+} & = & {\hat{x}}_{n}^{-}+K_{n}\left(z_{n}-H_{n}{\hat{x}}_{n}^{-}\right)\\ \Sigma_{{\hat{x}}_{n}^{+}} & = & \left(1-K_{n}H_{n}\right)\Sigma_{{\hat{x}}_{n}^{-}}{\left(1-K_{n}H_{n}\right)}^{T}+K_{n}\left(\Sigma_{z_{n}}\right)K_{n}^{T}. \end{array}$$

#### 3.2.3. Integer Ambiguity Fixing Using Prior Information on Baseline Coordinates

In this section, we exploit the integer property of ambiguities and describe the fixing of float ambiguities to integer ones. A joint fixing of ambiguities from *both* baselines is performed to exploit the correlation introduced by the use of a common receiver in both baselines. The integer ambiguities, baselines and pseudorange multipaths are determined such that the sum of squared residuals is minimized, i.e.,

(15) $$\begin{array}{c} \min_{\left\{N_{12},N_{12}\right\}\in Z,\;\;\left\{{\vec{b}}_{12}^{n},{\vec{b}}_{13}^{n},\Delta \rho_{{\mathrm{MP}}_{12}},\Delta \rho_{{\mathrm{MP}}_{13}}\right\}\in R\;}{∥\left(\begin{array}{c} {\hat{N}}_{12}\\ {\hat{N}}_{13} \end{array}\right)-\left(\begin{array}{c} N_{12}\\ N_{13} \end{array}\right)∥}_{\Sigma_{{\hat{N}}_{12},{\hat{N}}_{13}}^{-1}}^{2}\\ \;\;\;\;\;\;\;\;\;\;\;\;\;\;+{∥\left(\begin{array}{c} {\overset{ˇ}{\vec{b}}}_{12}^{n}\left(N_{12}\right)\\ {\overset{ˇ}{\vec{b}}}_{13}^{n}\left(N_{13}\right)\\ \Delta {\overset{ˇ}{\rho }}_{{\mathrm{MP}}_{12}}\left(N_{12}\right)\\ \Delta {\overset{ˇ}{\rho }}_{{\mathrm{MP}}_{13}}\left(N_{13}\right) \end{array}\right)-\left(\begin{array}{c} {\vec{b}}_{12}^{n}\\ {\vec{b}}_{13}^{n}\\ \Delta \rho_{{\mathrm{MP}}_{12}}\\ \Delta \rho_{{\mathrm{MP}}_{13}} \end{array}\right)∥}_{\Sigma_{{\overset{ˇ}{b}}_{12},{\overset{ˇ}{b}}_{13}}^{-1}}^{2}. \end{array}$$

An analytical optimization is not feasible due to the multi-dimensional integer parameters. Therefore, a sequential tree search is performed as suggested by Teunissen in [1] and Jonge and de Tiberius [9]. The ambiguity residuals $r_{N_{1r}^{kl}}:={\hat{N}}_{1r}^{kl}-N_{1r}^{kl}$ are expressed in terms of conditional ambiguities ${\hat{N}}_{1r}^{kl|1l,\ldots ,(k-1)l}$ using the triangular decomposition of the float ambiguity covariance matrix (see Blewitt [10]). The lower and upper bound of the search interval of ambiguity $N_{1r}^{kl}$ are derived by Jonge and de Tiberius in [9] as:

(16) $$\begin{array}{ccc} N_{1r}^{kl} & \geq & {\hat{N}}_{1r}^{kl|1l,\ldots (k-1)l}-\sigma_{{\hat{N}}_{1r}^{kl|1l,\ldots (k-1)l}}\cdot \sqrt{\chi^{2}-\sum_{j=1}^{k-1}\frac{{\left({\hat{N}}_{1r}^{jl|1l,\ldots (j-1)l}-N_{1r}^{jl|1l,\ldots (j-1)l}\right)}^{2}}{\sigma_{{\hat{N}}_{1r}^{jl|1l,\ldots (j-1)l}}^{2}}} \end{array}$$

(17) $$\begin{array}{ccc} N_{1r}^{kl} & \leq & {\hat{N}}_{1r}^{kl|1l,\ldots (k-1)l}+\sigma_{{\hat{N}}_{1r}^{kl|1l,\ldots (k-1)l}}\cdot \sqrt{\chi^{2}-\sum_{j=1}^{k-1}\frac{{\left({\hat{N}}_{1r}^{jl|1l,\ldots (j-1)l}-N_{1r}^{jl|1l,\ldots (j-1)l}\right)}^{2}}{\sigma_{{\hat{N}}_{1r}^{jl|1l,\ldots (j-1)l}}^{2}}}, \end{array}$$

with $\chi^{2}$ being the search space volume. A prior knowledge on the baselines is typically available in the body-fixed (b-) frame. The local (East-North-Up) navigation (n-) frame is related to the body-fixed frame via the attitude angles (roll φ, pitch θ and heading ψ), i.e., the baseline can be re-parameterized as

(18) $${\vec{b}}_{1r}^{n}=R_{b}^{n}\left(\varphi ,\theta ,\psi \right){\vec{b}}_{1r}^{b}\;\mathrm{with}\;R_{n}^{b}={\left(R_{b}^{n}\right)}^{-1}=R_{1}\left(\varphi \right)R_{2}\left(\theta \right)R_{3}\left(\psi \right).$$

The estimation of all three attitude angles requires at least three receivers spanning two baselines ${\vec{b}}_{12}$ and ${\vec{b}}_{13}$. Once baseline estimates are available in both frames, the rotation matrix $R_{b}^{n}\left(\varphi ,\theta ,\psi \right)$ can be determined using Wahba’s solution [11]. It minimizes the cost function

(19) $$C\left(\varphi ,\theta ,\psi \right)=\sum_{r=2}^{3}∥{\vec{b}}_{1r}^{\;n}-R_{b}^{n}\left(\varphi ,\theta ,\psi \right){\vec{b}}_{1r}^{\;b}{∥}^{2}.$$

Once the rotation matrix is found, the attitude angles can obtained from Equation (4):

(20) $$\begin{array}{ccc} \hat{\varphi } & = & \mathrm{atan}\left({\left(R_{n}^{b}\right)}_{1,2}/{\left(R_{n}^{b}\right)}_{1,1}\right),\\ \hat{\theta } & = & \mathrm{asin}\left({\left(-R_{n}^{b}\right)}_{1,3}\right),\\ \hat{\psi } & = & \mathrm{atan}\left({\left(R_{n}^{b}\right)}_{2,3}/{\left(R_{n}^{b}\right)}_{3,3}\right). \end{array}$$

We constrain the sequential tree search of inequality

(21) $${\hat{N}}_{1r}^{kl}-\sigma_{{\hat{N}}_{1r}^{kl}}\cdot \gamma^{kl}\leq N_{1r}^{kl}\leq {\hat{N}}_{1r}^{kl}+\sigma_{{\hat{N}}_{1r}^{kl}}\cdot \gamma^{kl}\;\forall \;k,$$

by integrating prior information on the baseline lengths and coordinates (provided in body-fixed frame), i.e., we consider only these integer candidates $N_{1r}^{kl}$, where the respective baseline estimate shows consistent baseline length and attitude information. This leads to the following additional requirements:

(22) $$\frac{{\left(∥{\overset{ˇ}{\vec{b}}}_{1r}^{\;n}\left(N_{1r}^{1l},\ldots ,N_{1r}^{kl}\right)∥-∥{\vec{b}}_{1r}^{\;b}∥\right)}^{2}}{\sigma_{{\overset{ˇ}{l}}_{1r}}^{2}+\sigma_{{\bar{l}}_{1r}}^{2}}\overset{!}{\leq }\gamma_{l_{1r}}^{2}\;\forall \;r\;\wedge ,$$

(23) $$\min_{\varphi ,\theta ,\psi }\left(\sum_{r=2}^{3}{∥{\overset{ˇ}{\vec{b}}}_{1r}^{\;n}\left(N_{1r}^{1l},\ldots ,N_{1r}^{kl}\right)-R_{b}^{n}\left(\varphi ,\theta ,\psi \right){\vec{b}}_{1r}^{\;b}∥}^{2}\right)\overset{!}{\leq }\gamma_{\varphi ,\theta ,\psi }^{2},$$

with the following notations:
${\overset{ˇ}{\vec{b}}}_{1r}^{\;n}\left(N_{1r}^{1l},\ldots N_{1r}^{kl}\right)$baseline estimate for partially fixed integer ambiguities,$\sigma_{{\overset{ˇ}{l}}_{1r}}^{2}$variance of length of baseline estimate assuming correct partial ambiguity fixing,$\sigma_{{\bar{l}}_{1r}}^{2}$variance of prior information on baseline length,$\gamma_{l}^{2}$upper bound on the squared normalized baseline length error,$\gamma_{\varphi ,\theta ,\psi }^{2}$upper bound on the sum of squared baseline residuals.


We derive the baseline estimate for partially fixed ambiguities: the double difference ambiguities are split into *float* ambiguities $N_{1r}^{1}$ and *fixed* ambiguities $N_{1r}^{2}$. As float and fixed double difference ambiguities in $N_{1r}$ are not sorted, the mapping of $N_{1r}^{1}$ and $N_{1r}^{2}$ to $N_{1r}$ is given by

(24) $$\begin{array}{ccc} \lambda N_{1r} & = & A_{1r}^{1}\left(\lambda \right)N_{1r}^{1}+A_{1r}^{2}\left(\lambda \right)N_{1r}^{2}, \end{array}$$

where $A_{1r}^{1}$ and $A_{1r}^{2}$ describe the mapping of float/integer ambiguity vectors $N_{1r}^{1}$ and $N_{1r}^{2}$ to $N_{1r}$. The ambiguity decomposition is included in the measurement model of Equation (10) being rewritten as

(25) $$\begin{array}{cc} & z_{n}=\underset{\bar{H}}{\underset{︸}{\left(\begin{array}{cc} H & 0\\ 0 & H\\ H & 0\\ 0 & H \end{array}\right)}}\left(\begin{array}{c} {\vec{b}}_{12}^{n}\left(t_{n}\right)\\ {\vec{b}}_{13}^{n}\left(t_{n}\right) \end{array}\right)+\underset{{\bar{A}}^{1}}{\underset{︸}{\left(\begin{array}{cc} A_{12}^{1}\left(\lambda \right) & 0\\ 0 & A_{13}^{1}\left(\lambda \right)\\ 0 & 0\\ 0 & 0 \end{array}\right)}}\left(\begin{array}{c} N_{12}^{1}\\ N_{13}^{1} \end{array}\right)+\underset{{\bar{A}}^{2}}{\underset{︸}{\left(\begin{array}{cc} A_{12}^{2}\left(\lambda \right) & 0\\ 0 & A_{13}^{2}\left(\lambda \right)\\ 0 & 0\\ 0 & 0 \end{array}\right)}}\left(\begin{array}{c} N_{12}^{2}\\ N_{13}^{2} \end{array}\right)\\ & \;\;\;\;+\eta_{z_{n}}. \end{array}.$$

The fixed ambiguities ${\overset{ˇ}{N}}_{1r}^{2}$, r∈{2,3} are subtracted from the measurements. Subsequently, a projection on the space orthogonal to ${\bar{A}}^{1}$ is applied to eliminate all non-baseline terms, i.e.,

(26) $$P_{{\bar{A}}^{1}}^{⊥}\left(z_{n}-{\bar{A}}^{2}\left(\begin{array}{c} {\overset{ˇ}{N}}_{12}^{2}\\ {\overset{ˇ}{N}}_{13}^{2} \end{array}\right)\right)=P_{{\bar{A}}^{1}}^{⊥}\left(\bar{H}\left(\begin{array}{c} {\vec{b}}_{12}^{n}\left(t_{n}\right)\\ {\vec{b}}_{13}^{n}\left(t_{n}\right) \end{array}\right)+\eta_{z_{n}}\right).$$

The notation is further simplified by defining $\bar{A}:=P_{{\bar{A}}^{1}}^{⊥}\bar{H}$. The least-squares estimate of both partially fixed baselines follows as

(27) $$\left(\begin{array}{c} {\overset{ˇ}{\vec{b}}}_{12}^{n}\left(t_{n}\right)\\ {\overset{ˇ}{\vec{b}}}_{13}^{n}\left(t_{n}\right) \end{array}\right)={\left({\bar{A}}^{T}\Sigma^{-1}\bar{A}\right)}^{-1}{\bar{A}}^{T}\Sigma^{-1}\left(z_{n}-{\bar{A}}^{2}\left(\begin{array}{c} {\overset{ˇ}{N}}_{12}^{2}\\ {\overset{ˇ}{N}}_{13}^{2} \end{array}\right)\right).$$

The accuracy of the baseline estimates depends on the number of fixed ambiguities: if less than three ambiguities are fixed per baseline, the pseudoranges are needed and the accuracy is limited by the pseudorange multipath. If at least three ambiguities are fixed per baseline, the baselines can be estimated solely with carrier phase measurements resulting in a much higher accuracy.

The efficiency of the ambiguity fixing is substantially improved by the integer decorrelation of Teunissen [1]. The integer decorrelation transformation *Z* is applied to the double difference ambiguities, i.e.,

(28) $$Z\left(\begin{array}{c} N_{12}\\ N_{13} \end{array}\right)=Z^{1}\left(\begin{array}{c} N_{12}^{1}\\ N_{13}^{1} \end{array}\right)+Z^{2}\left(\begin{array}{c} N_{12}^{2}\\ N_{13}^{2} \end{array}\right):=\left(\begin{array}{c} {\tilde{N}}_{12}^{1}\\ {\tilde{N}}_{13}^{1} \end{array}\right)+\left(\begin{array}{c} {\tilde{N}}_{12}^{2}\\ {\tilde{N}}_{13}^{2} \end{array}\right).$$

The integer decorrelation transformation is invertible and preserves the integer property, i.e., the uncorrelated double difference ambiguities can be obtained from the decorrelated ones by

(29) $$\left(\begin{array}{c} N_{12}\\ N_{13} \end{array}\right)=Z^{-1}\left(\left(\begin{array}{c} {\tilde{N}}_{12}^{1}\\ {\tilde{N}}_{13}^{1} \end{array}\right)+\left(\begin{array}{c} {\tilde{N}}_{12}^{2}\\ {\tilde{N}}_{13}^{2} \end{array}\right)\right).$$

The decorrelated fixed ambiguities ${\tilde{N}}_{1r}^{1}$ and float ambiguities ${\tilde{N}}_{1r}^{2}$ are introduced in the measurement model of Equation (25), which is rewritten as

(30) $$\left(z_{n}-\underset{\tilde{A}}{\underset{︸}{A\left(\lambda \right)Z^{-1}}}\left(\begin{array}{c} {\overset{ˇ}{\tilde{N}}}_{12}^{2}\\ {\overset{ˇ}{\tilde{N}}}_{13}^{2} \end{array}\right)\right)=\bar{H}\left(\begin{array}{c} {\vec{b}}_{12}^{n}\left(t_{n}\right)\\ {\vec{b}}_{13}^{n}\left(t_{n}\right) \end{array}\right)+\underset{\tilde{A}}{\underset{︸}{A\left(\lambda \right)Z^{-1}}}\left(\begin{array}{c} {\tilde{N}}_{12}^{1}\\ {\tilde{N}}_{13}^{1} \end{array}\right)+\eta_{z_{n}}.$$

The decorrelated *float* ambiguities are eliminated by a projection on the space orthogonal of $\tilde{A}$. The least-squares baseline estimates for partially fixed decorrelated ambiguities follows as

(31) $$\left(\begin{array}{c} {\overset{ˇ}{\vec{b}}}_{12}^{n}\left(t_{n}\right)\\ {\overset{ˇ}{\vec{b}}}_{13}^{n}\left(t_{n}\right) \end{array}\right)={\left({\bar{\tilde{A}}}^{T}\Sigma^{-1}\bar{\tilde{A}}\right)}^{-1}{\bar{\tilde{A}}}^{T}\Sigma^{-1}\left(z_{n}-\tilde{A}\left(\begin{array}{c} {\overset{ˇ}{\tilde{N}}}_{12}^{2}\\ {\overset{ˇ}{\tilde{N}}}_{13}^{2} \end{array}\right)\right),$$

with $\bar{\tilde{A}}=P_{\tilde{A}}^{⊥}\bar{H}$.

The baseline estimates are used to constrain the search interval in Equations (22) and (23). The reduction of search space enables a much more reliable ambiguity fixing.

## 4. Fast Initialization of GNSS Attitude Ambiguity Fixing with Calibrated Magnetometers

In this section, we describe the benefit of magnetometer-based attitude information for kinematic GNSS-based attitude determination.

In a first step, we derive the attitude from calibrated magnetic flux measurements. It is assumed that precise estimates of the sensor bias ${\hat{b}}_{m^{s}}$ and of the magnetic flux strength ${\hat{m}}^{g}$ are available, e.g., using a previous calibration. In this case, the attitude information is obtained from the magnetic flux measurements by minimizing the sum of squared residuals, i.e.,

(32) $$\begin{array}{c} \min_{\varphi \left(t_{j}\right),\theta \left(t_{j}\right),\psi \left(t_{j}\right)}∥m^{s}\left(t_{j}\right)-{\hat{b}}_{m^{s}}-R_{b}^{s}\left(\Delta \hat{\varphi },\Delta \hat{\theta },\Delta \hat{\psi }\right)\cdot R_{n}^{b}\left(\varphi \left(t_{j}\right),\theta \left(t_{j}\right),\psi \left(t_{j}\right)\right)\cdot R_{g}^{n}\left(\Delta \hat{\nu }\right)\cdot {\hat{m}}^{g}{∥}^{2}\\ =\min_{\varphi \left(t_{j}\right),\theta \left(t_{j}\right),\psi \left(t_{j}\right)}∥R_{s}^{b}\left(\Delta \hat{\varphi },\Delta \hat{\theta },\Delta \hat{\psi }\right)\cdot \left(m^{s}\left(t_{j}\right)-{\hat{b}}_{m^{s}}\right)-R_{n}^{b}\left(\varphi \left(t_{j}\right),\theta \left(t_{j}\right),\psi \left(t_{j}\right)\right)R_{g}^{n}\left(\Delta \hat{\nu }\right)\cdot {\hat{m}}^{g}{∥}^{2}. \end{array}$$

This minimization refers to the well-known Wahba’s problem. Its solution is provided in [11].

In a second step, we use this *attitude* information as *prior knowledge* in the GNSS-based attitude determination. The GNSS carrier phase ambiguities are again determined with a sequential tree search as described in the previous section.

However, the second constraint (23) on the attitude substantially simplifies as the attitude angles are *known* from the calibrated magnetometer. Thus, the minimization and Wahba’s solution [11] are no longer needed and Equation (23) simplifies to

(33) $$\sum_{r=2}^{3}{∥{\overset{ˇ}{\vec{b}}}_{1r}^{\;n}\left(N_{1r}^{1l},\ldots ,N_{1r}^{kl}\right)-R_{b}^{n}\left(\hat{\varphi },\hat{\theta },\hat{\psi }\right){\vec{b}}_{1r}^{\;b}∥}^{2}\overset{!}{\leq }\gamma_{\varphi ,\theta ,\psi }^{2}.$$

**Figure 1:**
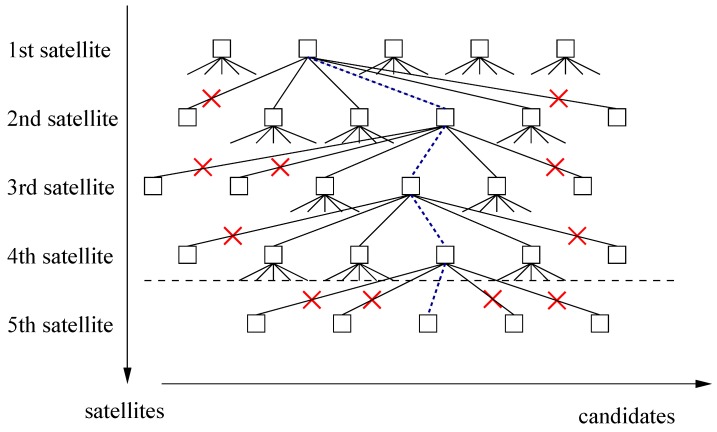
Integer ambiguity fixing: sequential tree search with attitude constraints.

Figure 1 visualizes the constrained tree search. The integer candidates are determined sequentially for the satellites, whereas the integer candidates of each satellite are conditioned on the integer ambiguity candidates of the previous satellites. There are typically multiple integer candidates for each satellite. However, several integer candidates can be dropped due to inconsistent baseline length and attitude information, which reduces the number of branches to be searched.

### Analysis of Benefit of Magnetometer-Based Attitude Information for GNSS Integer Ambiguity Fixing

In this section, we analyze the benefit of magnetometer-based attitude information for GNSS integer ambiguity fixing. Galileo double difference carrier phase and pseudorange measurements are simulated according to Equations (8) and (9) with the settings of Table 1.

The integer ambiguity estimation is performed in three steps:

- Estimation of float solution of baselines and ambiguities by least-squares estimation using single epoch measurements,
- Normalization of baseline estimates with prior information on baseline length and respective adjustment of float ambiguities,
- Integer ambiguity fixing with sequential tree search and integer decorrelation using magnetometer-based attitude information and baseline length prior information.

**Figure 2:**
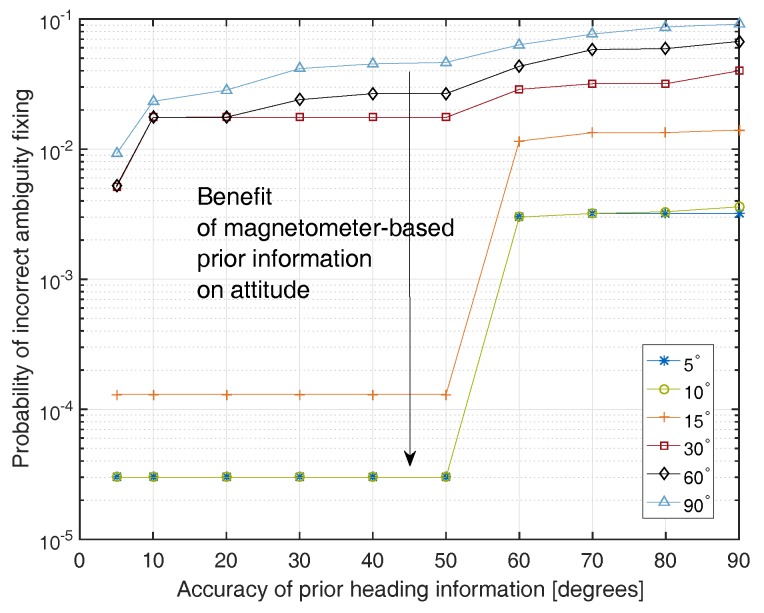
Benefit of magnetometer-based prior attitude information for single epoch integer ambiguity fixing: the accuracy of the prior heading information is shown on the *x*-axis and the accuracy of the prior pitch information is provided in the legend.

Figure 2 shows the probability of incorrect integer ambiguity fixing as a function of the accuracy of the magnetometer-based heading and pitch angle accuracies. We can observe that the probability of incorrect integer ambiguity fixing is reduced by **several orders of magnitude** by the magnetometer based heading/pitch angle with an accuracy of $10^{\circ }$.

**Figure 3:**
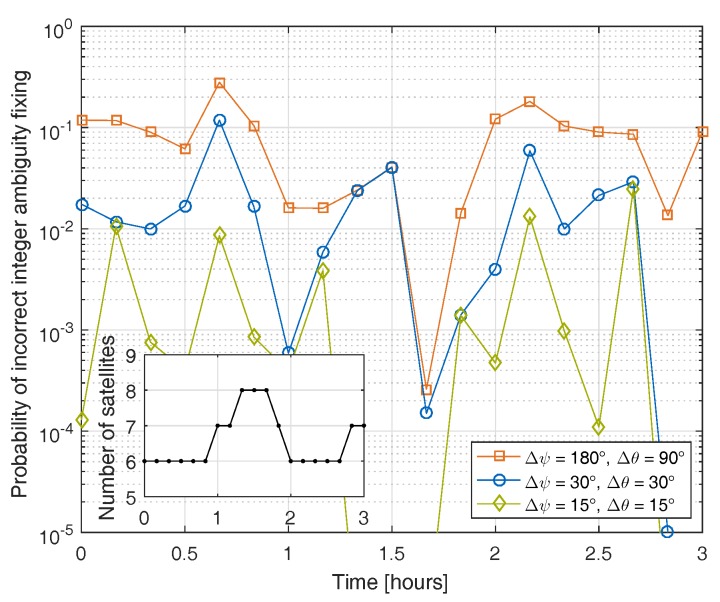
Benefit of magnetometer-based prior attitude information for single epoch integer ambiguity fixing: the probability varies over time due to the changing satellite constellation.

Figure 3 shows the probability of incorrect single epoch integer ambiguity fixing with attitude prior information over time. One can observe that the probability varies with the change of the satellite constellation, and can be significantly reduced by the magnetometer-based attitude information.

## 5. Measurement Results

This section describes the verification of the proposed magnetometer calibration and the achievable heading accuracy in a test drive using a tightly coupled GNSS/INS reference system. The method and results differ in the following aspects from our earlier paper [5]:

- Calibration with Multi-GNSS (GPS + GLONASS)/INS tightly coupled attitude information instead of GPS-only attitude estimate, enabling higher reliability due to inertial sensors and faster calibration due to higher update rate,
- Use of three instead of two GNSS receivers for full 3D attitude information,
- Estimation of 3D magnetic flux in North-East-Down frame instead of 1D magnetic flux in the North-only direction, enabling use also in areas with systematic distortions of magnetic field and/or close to magnetic poles,
- Use of the newest sensor generation: LEA M8T Multi-GNSS receiver of u-blox (Thalwil, Switzerland), Taoglas AGGP.35F dual-band GNSS antenna (Enniscorthy, Ireland), and MPU 9250 inertial sensor (San Jose, CA, USA).

The MPU 9250 [13] includes a three-axis silicon monolithic Hall-effect magnetic sensor with magnetic concentrator. The magnetic flux measurements are provided with a resolution of 0.6 μT. Three multi-sensor modules were mounted on the roof of a vehicle, whereas each multi-sensor module included the above sensors. The inertial sensor provides 3D acceleration, 3D angular rate and 3D *magnetometer* measurements in the sensor-fixed frame with an update rate of 100 Hz. The distances between the GNSS antennas were around 1 m. The Multi-GNSS (GPS + GLONASS)/INS tightly coupled attitude determination included some pre-processing (synchronization, cycle slip correction [14,15] calibration of inertial sensor biases) and carrier phase integer ambiguity fixing.

**Figure 4:**
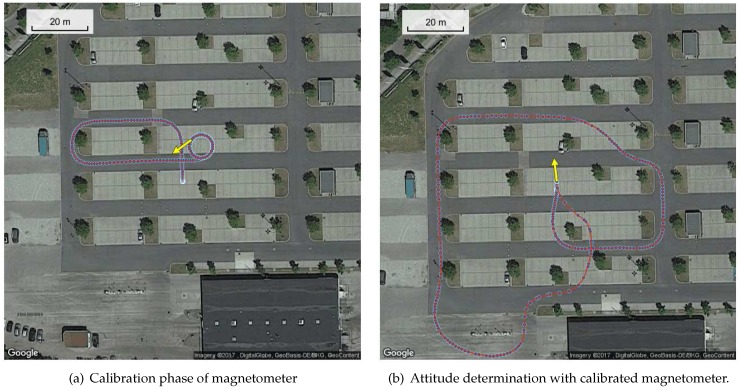
Maps with track of vehicle, split into two processing steps.

Figure 4 shows the track of the test drive. It was split into two phases, i.e., a first phase for calibrating the magnetometer, and a second phase for testing the calibration of the magnetometer. In both phases, the track includes rotational dynamics.

**Figure 5:**
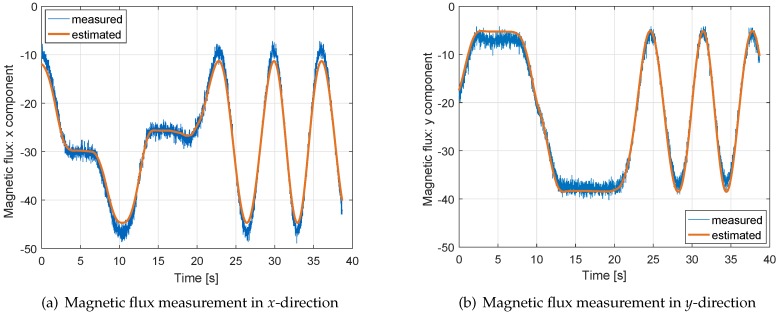
Magnetic flux measurements in sensor-fixed frames: the measured magnetic flux in *x*- and *y*- directions depends on the heading and is quite noisy. The least-squares estimate of the magnetic flux is much less noisy, as the estimation combines the measurements from 40 s to determine the 3D magnitude of the magnetic flux and the 3D biases.

Figure 5 shows the accuracy of the magnetic flux measurements and of our measurement model, i.e., the raw measurements are compared to estimated measurements derived from the least-squares estimate of the magnetic flux in the North-East-Down frame and the sensor biases. Obviously, both tracks fit quite well. The systematic variations over time in both subfigures are caused by changes in the attitude (especially heading). The estimated measurements are less noisy as the precise Multi-GNSS/INS tightly coupled attitude is used and as constant magnitudes of the magnetic field in North-East-Down directions are assumed over the considered period of 40 s.

**Figure 6:**
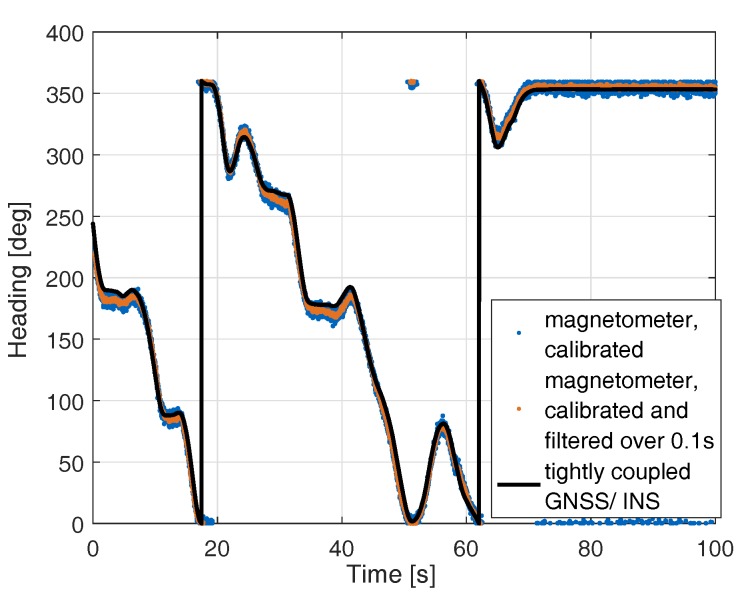
Heading determination with calibrated magnetometer in comparison to tightly-coupled GNSS/INS heading: the latter one has an accuracy of 0.25 degrees and serves as a reference. As the magnetometer-based heading is noisy, a filtered version with a time constant of 0.1 s is also shown.

**Figure 7:**
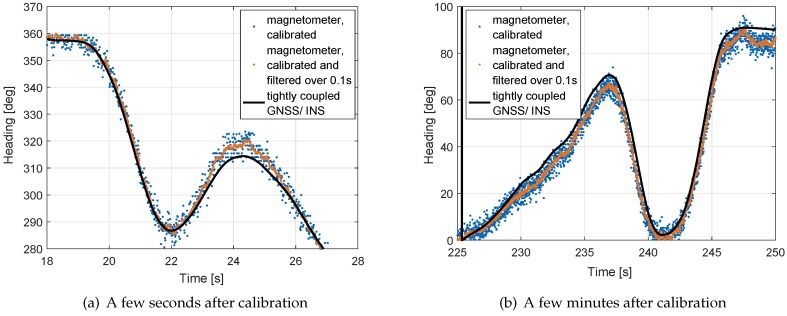
Detailed analysis of heading performance for two sections with moderate to high rotational dynamics: the filtered magnetometer-based heading deviates by less than 10 degrees from the GNSS/INS tightly coupled heading.

Figure 6 shows the heading using calibrated magnetometer measurements in comparison with the tightly-coupled Multi-GNSS/INS heading. The latter one has an accuracy of 0.25 degrees and serves as reference. The figure also includes a filtered, calibrated magnetometer heading with a time constant of 0.1 s to reduce the noise of the magnetometer. The filtered magnetometer-based heading deviates by less than 10 degrees from the GNSS/INS tightly coupled heading as shown in the enlarged sections in Figure 7.

## 6. Conclusions

In this paper, a method for calibration of magnetic field sensors with three Global Navigation Satellite System (GNSS) receivers was provided. A GNSS-based attitude information was obtained from the relative positions between the GNSS receivers. The relative positions were jointly estimated with the carrier phase integer ambiguities and pseudorange multipath errors. The latter ones were considered as state parameters to exploit the spatial and temporal correlations of multipath. The reliability of carrier phase integer ambiguity fixing was improved by integration of prior information on the baseline coordinates provided in a local body-frame.

The attitude information of the calibrated magnetometer was also integrated into the sequential tree search of ambiguity fixing, i.e., integer candidates with inconsistent attitude information were disregarded. It was shown that a heading and pitch angle accuracy of 10 degrees are sufficient to reduce the probability of incorrect instantaneous ambiguity fixing by several orders of magnitude.

Finally, the proposed method was tested with three low-cost Multi-GNSS receivers and a magnetic field sensor in a test drive. A tightly coupled Multi-GNSS/INS system served as a reference. The measurement results showed that the calibrated and filtered heading differed by less than $10^{\circ }$ from the reference heading.

## Figures and Tables

**Table 1:** Simulated Galileo double difference measurements.

simulated measurements	single frequency double difference
	pseudoranges and carrier phase measurements
	on L1 ($f_{c}=1575.42$ MHz) of 27 Galileo satellites
	using nominal Walker constellation [12]
	(satellite altitude: 23,222 km, orbital inclination: $56^{\circ }$)
receiver position	longitude $\lambda =11.568578^{\circ }$ E, latitude $\varphi =48.150889^{\circ }$ N
baseline vector	length of 1 m, random attitude angles
noise statistics	phase noise: $\sigma_{\varphi }=2$ mm
	code noise: $\;\;\sigma_{\rho }=2$ m including multipath
accuracy of prior information on baseline length	$\sigma_{{\bar{l}}_{1r}}=2$ cm
accuracy of magnetometer based attitude information	variable accuracies for both heading and pitch angles
